# Oocytes, embryos and pluripotent stem cells from a biomedical perspective

**DOI:** 10.21451/1984-3143-AR2019-0054

**Published:** 2019-10-23

**Authors:** Poul Hyttel, Laís Vicari de Figueiredo Pessôa, Jan Bojsen-Møller Secher, Katarina Stoklund Dittlau, Kristine Freude, Vanessa J Hall, Trudee Fair, Remmy John Assey, Jozef Laurincik, Henrik Callesen, Torben Greve, Lotte Björg Stroebech

**Affiliations:** 1 Department of Veterinary and Animal Sciences, University of Copenhagen, Denmark.; 2 Department of Veterinary Clinical Sciences, University of Copenhagen, Denmark.; 3 KU Leuven – University of Leuven, Department of Neurosciences, Experimental Neurology, and Leuven Brain Institute (LBI), Leuven, Belgium.; 4 VIB, Center for Brain & Disease Research, Laboratory of Neurobiology, Leuven, Belgium.; 5 School of Agriculture and Food Science, University College Dublin, Belfield, Dublin 4, Ireland.; 6 Department of Anatomy and Pathology, Sokoine University of Agriculture, Tanzania.; 7 Constantine the Philosopher University in Nitra, Nitra, Slovakia.; 8 The Czech Academy of Sciences, Institute of Animal Physiology and Genetics, Liběchov, Czech Republic.; 9 Department of Animal Science, Aarhus University, Tjele, Denmark.; 10 Nøddehaven, Værløse.

**Keywords:** embryonic stem cells, induced pluripotent stem cells, *in vitro* fertilization, Alzheimer’s disease, dementia

## Abstract

The veterinary and animal science professions are rapidly developing and their inherent and historical connection to agriculture is challenged by more biomedical and medical directions of research. While some consider this development as a risk of losing identity, it may also be seen as an opportunity for developing further and more sophisticated competences that may ultimately feed back to veterinary and animal science in a synergistic way. The present review describes how agriculture-related studies on bovine *in vitro* embryo production through studies of putative bovine and porcine embryonic stem cells led the way to more sophisticated studies of human induced pluripotent stem cells (iPSCs) using e.g. gene editing for modeling of neurodegeneration in man. However, instead of being a blind diversion from veterinary and animal science into medicine, these advanced studies of human iPSC-derived neurons build a set of competences that allowed us, in a more competent way, to focus on novel aspects of more veterinary and agricultural relevance in the form of porcine and canine iPSCs. These types of animal stem cells are of biomedical importance for modeling of iPSC-based therapy in man, but in particular the canine iPSCs are also important for understanding and modeling canine diseases, as e.g. canine cognitive dysfunction, for the benefit and therapy of dogs.

## Introduction

The veterinary and animal science professions are rapidly developing in a shifting scientific environment. Worldwide institutional reorganizations towards larger entities result in absorption of veterinary and animal science faculties into broader entities with a focus on life, biomedical and medical sciences. While this development has a range of advantages creating novel scientifically rewarding collaborative landscapes it also challenges the conventional identity of the veterinary and animal science professions and their inherent and historical connection to agriculture. Consequently, the focus of veterinary and animal sciences has been extended with a major biomedical and even medical dimension; a development which is also sparked by a shift in funding opportunities with biomedicine and medicine having higher leverage than agriculture. Some consider the gradual increase in biomedical and medical focus, at the expense of agricultural attention, a risk. On the other hand, this development gives more room for investigating the complex area of “One Health” and may also give veterinarians and animal scientists access to new sets of competences, that may, in a synergistic and constructive way, feedback to more core classical veterinary and animal science.

It is fair to say that the biomedical and medical trend in science cannot be rejected and should be contemplated as an opportunity for contemporary development of the veterinary and animal science professions. It is the focus of this review to present a scientific development where research in assisted reproductive technologies (ARTs) and embryonic stem cells (ESCs) in the large domestic species has given opportunities for establishing a stem cell center of excellence in neurology focusing on human induced pluripotent stem cell (iPSC)-models for neurodegeneration and, finally, how the competences gained through these medical activities allowed for investigations of porcine and canine iPSCs feeding positively back to veterinary and animal science (Fig.1).

**Figure:**
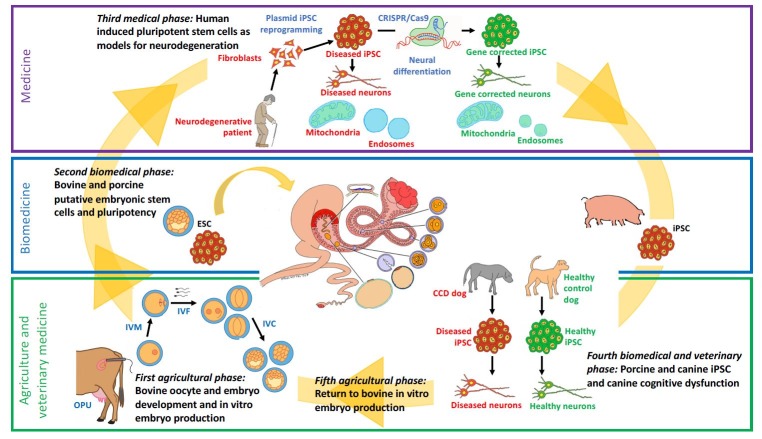
1. The progression of research activities moving from agricultural and veterinary medicine through biomedicine and medicine and feeding back to agricultural and veterinary medicine. OPU: Ultrasound-guided ovum pickup; IVM: *In vitro* maturation; IVF: *In vitro* fertilization; IVC: *In vitro* culture.

## First agricultural phase: Bovine oocyte and embryo development and *in vitro* embryo production

### In vitro embryo production in cattle

Over the past 40 years there has been a gradual agricultural implementation of novel ARTs in large animal husbandry with particular focus on cattle (Greve and Callesen, 2005; Lonergan, 2007). Major components of this development have been the development and refinement of the multiple ovulation and embryo transfer (MOET), including cryopreservation of blastocysts, and of *in vitro* fertilization (IVF) culminating with the birth of the first IVF calf in 1981 (Brackett *et al*., 1982). Whereas contemporary *in vitro* production (IVP) of embryos in cattle includes *in vitro* oocyte maturation, *in vitro* fertilization and *in vitro* culture of the resultant embryos to the blastocyst stage, Brackett and colleagues flushed *in vivo* matured oocytes from the oviducts, performed IVF and transferred a 4-cell stage back to the oviduct. In 1987, the first European IVF calf, now resulting from *in vitro* oocyte maturation, was born in Copenhagen (Xu *et al*., 1987) where we invested great efforts in fundamental investigations of oocyte maturation and fertilization (Hyttel *et al*., 1986a; Hyttel *et al*., 1986b; Hyttel *et al*., 1988a; Hyttel *et al*., 1988b).

In parallel, the quest for defining optimal culture conditions allowing for the development of bovine zygotes to blastocysts took place including focus on coculture systems (Edwards *et al*., 1997), media composition (Holm *et al*., 1999) as well as the physical design of the culture platforms (Vajta *et al*., 2008; Smith *et al*., 2012). These efforts all became extremely relevant in the light of the astonishing adverse effects of improper *in vitro* culture conditions that were reported in 1997, and which coined the term Large Offspring Syndrome (LOS) (Kruip and den Daas, 1997). The risk of LOS caused severe drawbacks for the technologies, and in Denmark the practical implementation of *in vitro* embryo production in cattle breeding was abandoned mainly for this reason. Refined serum-free culture conditions, based on BSA supplementation, have now been developed allowing for improved fetal development and calving (George *et al*., 2008), and in 2013 an entire serum-free ready-to-use media suite for all the steps, maturation, fertilization and culture, was made commercially available by IVF Bioscience, UK, combining synthetic serum replacements and BSA. Finally, the combination of IVP and ultrasound-guided ovum pickup (OPU) has allowed for more sophisticated practical implementation of IVP in cattle breeding, and the year of 2018 became a turning point as the numbers of transferred bovine IVP embryos for the first time officially exceeded that of their *in vivo*-derived counterparts. According to the numbers collected by the IETS, almost 1.5 million (1,487,343) bovine embryos were produced by MOET or IVP worldwide in 2017 and two thirds (almost 1 million) were derived by IVP (Viana, 2018).

In order to pave the way for successful IVP of bovine embryos, we undertook a series of fundamental studies of oocyte development, fertilization and initial embryonic development in cattle, which are summarized in the following. Hence, we have characterized oocyte development and maturation, fertilization and initial embryonic development in cattle extensively by transmission electron microscopy (TEM).

The basic ultrastructure of the oocyte is generated during its growth phase in the primordial to the tertiary follicle. When the tertiary follicles in a cohort reach a diameter of about 3-5 mm in cattle, one dominant follicle is selected, and the structure of the oocyte in this particular follicle is modified during a process that may be referred to as capacitation or pre-maturation. The estrous cycle in cattle generally comprises 2 or 3 follicular waves, and the dominant follicle of the last wave becomes ovulatory. In the ovulatory follicle the oocyte undergoes a final maturation during an approximately 24 hour period between the peak of the LH-surge and ovulation.

### Oocyte growth in cattle

During the growth of the bovine oocyte, the inside zona pellucida diameter of the gamete increases from less than 30 μm in the quiescent primordial follicle to more than 120 μm in the tertiary follicle. We have carefully characterized the ultrastructure, transcriptional activity and developmental competence of bovine oocytes in relation to the sequential stages of follicular development (Fair *et al*., 1996; Fair *et al*., 1997a; Fair *et al*., 1997b).

In the quiescent **primordial follicle** gap and intermediate junctions are present between adjacent granulosa cells, whereas exclusively intermediate junctions are seen between the granulosa cells and the oocyte. The transcriptionally quiescent nucleus of the oocyte, i.e. the germinal vesicle, occupies a central or slightly off center position and the organelles are concentrated in the perinuclear region. The **primary follicle** occasionally exhibits small portions of zona pellucida substance between the cuboidal granulosa cells and the oocyte. The continued zona-formation in the **secondary follicle** is associated with the embedding of granulosa cell processes and erect oocyte microvilli into the zona pellucida, and gap junctions are established between the granulosa cell processes and the oocyte. The oocyte nucleoli develop into a fibrillo-granular appearance and transcription is initiated. The oocyte in the **small tertiary follicle** up to about 1 mm in diameter exhibits a complete zona pellucida traversed by numerous cumulus cell projections forming gap and intermediate junctions to the oocyte. Clusters of cortical granules are numerous. The oocyte nucleoli are typical fibrillo-granular and transcription abundant. In the **larger tertiary follicles** the oocyte ultrastructure may be classified according to the inside zona pellucida diameter of the cell. In oocytes <100 μm the particular hooded mitochondria, unique to ruminants, are observed for the first time. Oocytes from 100 to 110 μm in diameter typically display formation of a perivitelline space, the process of which is associated with the release of the previously embedded microvilli from the zona pellucida. The oocyte nucleus is displaced towards the periphery as are Golgi complexes and mitochondria, amongst which the hooded form becomes more numerous. The fibrillar centers of the nucleoli have typically migrated towards the nucleolar periphery and transcription is decreased. Oocytes from 110 to 120 μm typically present a well-developed perivitelline space and a peripherally located nucleus. The process of nucleolar inactivation has proceeded leaving the nucleolus to consist of a spherical nucleolar remnant with a fibrillar center attached. At a diameter of 120 μm, the oocyte has completed the growth phase and achieved the ultrastructure characterizing the fully developed gamete.

Interestingly, the oocyte achieves the competence to complete meiotic maturation to metaphase II *in vitro* at a diameter of about 110 μm coinciding with the de-activation of its transcriptional machinery, indicating that the necessary compartment of proteins and mRNAs has been formed at this stage of development.

### Oocyte capacitation or pre-maturation in cattle

Further, we have carefully mapped the ultrastructural development of bovine oocytes in the dominant vs. the subordinate follicles (Assey *et al*., 1994a). With the growth of the dominant follicle, the ultrastructure of the fully grown oocyte is modified during its so-called capacitation or pre-maturation. During the days approaching the regression of the corpus luteum, i.e. the final period of the luteal phase, the cortical granule clusters are dislocated to more superficial locations and some granules migrate to solitary positions along the oolemma. During the period between luteolysis and the LH-surge individual cumulus cells exhibit elongation and some of the cumulus cell process endings are retracted to a more superficial location on the surface of the oolemma. Also, the oocyte nuclear envelope becomes undulating, especially in the regions facing the zona pellucida, and the nucleolar remnant displays vacuolization. Both of these phenomena are presumably related to the subsequent breakdown of the oocyte nucleus, i.e. germinal vesicle breakdown (GVBD). There are indications that the competence of the oocyte to produce blastocysts *in vitro* increases with completion of capacitation or pre-maturation in the dominant follicle (Hendriksen *et al*., 2000). Superovulation with exogenous gonadotropins may have an adverse effect on this process as indicated by a lack of at least the vacuolization of the nucleolar remnant (Assey *et al*., 1994b).

### Oocyte maturation in cattle

The maturation of the oocyte, which in cattle occurs during the approximately 24 hour period from the LH-peak to ovulation, comprises the progression of meiosis from the diplotene stage of prophase I to metaphase II accompanied by a series of ultrastructural and molecular changes in the ooplasm. The ultrastructural changes have been described in detail in relation to the time of the LH-peak in unstimulated (Kruip *et al*., 1983) as well as gonadotropin stimulated cattle (Hyttel *et al*., 1986a). The breakdown of the oocyte nucleus (GVBD) occurs 9 to 12 hours after the LH-peak when the nuclear envelope becomes extremely undulating, the chromatin condenses, the nucleolar remnant is dissolved and there is a gradual decoupling of the cumulus cell endings from the oocyte (Hyttel, 1987). At about 15 and 20 hours after the LH-peak most oocytes have reached metaphase I and II, respectively, and the first polar body is abstricted. During the last hours of maturation, lipid droplets and mitochondria attain a more central location in the ooplasm and the cortical granules migrate to solitary positions along the oolemma. The peripheral migration of the cortical granules appears to be compromised to a certain degree during oocyte maturation *in vitro* (Hyttel *et al*., 1986b).

Growing and dominant follicles are capable of maintaining oocyte meiosis arrested at the diplotene stage of prophase I. However, numerous subordinate tertiary follicles undergo atresia. Interestingly, such atretic follicles may lose the ability to retain the oocyte in meiotic arrest. Hence, oocytes in atretic follicles may display different stages of meiotic maturation; even reaching metaphase II (Assey *et al*., 1994a). Through the described phases of growth, capacitation and maturation, the oocyte has now acquired the ultrastructural architecture for sustaining fertilization and initial embryonic development.

### Fertilization and development of the zygote in cattle

The ultrastructure of bovine fertilization has precisely been described in relation to the estimated time of ovulation as determined by timing of the LH-peak in gonadotropin stimulated cows (Hyttel, *et al*., 1988a), and bovine *in vitro* fertilization have added to this understanding (Hyttel *et al*., 1988b; Hyttel *et al*., 1988c).

Upon acrosome reaction and penetration of the zona pellucida, the oocyte microvilli contact the equatorial segment of the sperm head where fusion between the two gametes initially occurs resulting in oocyte activation and cortical granule exocytosis establishing the block against polyspermic fertilization. With a correct Greek term, gamete fusion is termed syngamy; a term that erroneously is also widely used for the apposition of the pronuclei (see later). Within the first 2-3 hours after ovulation, the paternal chromatin is denuded from its membrane coverings and decondensed. In parallel, the maternal chromatin is advancing through anaphase and telophase II forming the second polar body. Pronucleus formation is initiated with smooth endoplasmic reticulum (SER) moving towards both the paternal and maternal chromatin to form nuclear envelope. About 4 hours after ovulation, the two sets of chromatin are completely surrounded by nuclear envelopes. The midpiece of the sperm tail remain spatially associated with the paternal pronucleus. Subsequently, the pronuclei swell to their characteristic spherical appearance accompanied by chromatin decondensation, and about 10 hours post ovulation most zygotes exhibit spherical pronuclei (Laurincik *et al*., 1998). Along with this process, so-called nucleolus precursor bodies, very similar to the oocyte nucleolar remnant, which later act as enucleation sites for nucleolus formation, are formed in the pronuclei (Laurincik *et al*., 1996). The precursor bodies are not active in rRNA transcription and ribosome formation. The two pronuclei migrate to a close apposition, and about 14 hours after ovulation most zygotes exhibit apposed pronuclei. The S-phase of the first post-fertilization cell cycle takes place 12-19 hours after ovulation (Laurincik *et al*., 1994). Upon pronuclear apposition, pronounced undulations of the nuclear envelopes of the pronuclei are seen in the apposed regions probably preparing for breakdown of the envelopes, which is seen at about 24 hours after ovulation. This process is often referred to as synkaryosis, but it should be emphasized that the two pronuclei do not fuse, but undergo dissolution of the nuclear envelopes similar to the one seen at the breakdown of the oocyte nucleus (GVBD) at resumption of oocyte meiosis. Immediately after synkaryosis, karyokinesis and cytokinesis proceed resulting in the formation of two daughter nuclei enclosed in each their blastomere.

### Pre-hatching embryonic development in cattle

Along with the initial cleavages, the embryonic genome is gradually activated during the so-called maternal-embryonic transition. Thus, a low rate of transcription of the embryonic genome has been detected as early as during the 1st, i.e. the zygote (Hay-Schmidt *et al*., 2001), and 2nd post-fertilization cell cycles (Hyttel *et al*., 1996; Viuff *et al*., 1996), and during the 4th cell cycle a major transcriptional activation occurs (Camous *et al*., 1986).

A number of other researchers have contributed to the understanding of the general embryonic ultrastructure based on either *in vivo* or *in vitro* developed embryos (Mohr and Trounson, 1981; Camous *et al*., 1986; Betteridge and Fléchon, 1988; King *et al*., 1988; Kopecný *et al*., 1989; Abe *et al*., 1999; Laurincik *et al*., 2000; Laurincik *et al*., 2003).

Early during the second cell cycle, i.e. the 2-cell stage, nucleolus precursor bodies resembling those described for the pronuclei are established in the nuclei. Hence, functional nucleoli are lacking and protein synthesis must be based on the ribosome pool inherited from the oocyte. Early during the third and fourth cell cycle, i.e. the tentative 4- and 8-cell stages, respectively, nucleolus precursor bodies resembling those from the previous cell cycles are again established. During the fourth cell cycle, however, the nucleolus precursor bodies develop into fibrillo-granular nucleoli displaying the typical components of actively ribosome-synthesizing nucleoli: Fibrillar centers, dense fibrillar component and granular component. The development of the nucleoli is a prerequisite for continued embryonic development and is a sensitive marker for the normality of this process. Abundant activation of embryonic transcription during the fourth cell cycle allows for the first cell differentiation and lineage commitments. External cells become connected by tight junctions while internal cells are only connected by focal membrane contacts. Mitochondria of the hooded form, which were established back during the development of the oocyte in the early tertiary follicle, become fewer, and elongated types with transverse cristae become more numerous.

The competences gained by our studies of oocyte maturation, fertilization and initial embryonic development in cattle allowed us to move into the stem cell area for creating novel potentials in agriculture and biomedicine.

## Second biomedical phase: Bovine and porcine putative embryonic stem cells and pluripotency

### Embryonic stem cells

Mouse embryonic stem cells (ESCs) were derived in 1981 (Evans and Kaufman, 1981; Martin, 1981) and paved the way for production of genetically modified mice (Thomas and Capecchi, 1987). Along with this development, an interest emerged in investigating the potentials for genomic modifications of the large domestic species for production, health, environmental and biomedical purposes.

Further studies of murine ESCs revealed that there are distinct states of pluripotency (naïve and primed) that differ both morphologically and functionally (De Los Angeles *et al*., 2012). Naïve murine pluripotent stem cells are derived from the inner cell mass (ICM) or early epiblast cells, proliferate in culture as packed dome-like colonies, are maintained in the undifferentiated state by LIF and BMP4 signaling, readily contribute to germline transmitting chimeric embryos, maintain two active X chromosomes (in female cells) and are relatively resistant to differentiation into primordial germ cells (PGCs) and extra-embryonic lineages (Kuijk *et al*., 2011). In contrast, primed pluripotent stem cells are derived from the epiblast of post-hatching murine blastocysts, are termed epiblast stem cells (EpiSCs), are molecularly and epigenetically different from murine ESCs (Brons *et al*., 2007; Tesar *et al*., 2007), have a more flattened colony morphology, depend on bFGF or TGFa/activin signaling, exhibit a limited ability to contribute to chimeras and have undergone X-chromosome inactivation (Brons *et al*., 2007). Human ESCs were first derived in 1998 (Thomson *et al*., 1998) and, surprisingly, they exhibit characteristics more like those of primed murine EpiSCs than their naïve murine ESC counterparts (Thomson *et al*., 1998).

The potentials of murine ESCs for the generation of transgenic mice sparked an interest in deriving ESCs in the large domestic species including activities in our laboratories focusing on cattle and pig. We and many others attempted to derive bovine ESCs (for review, see Ezashi *et al*., 2016) from different developmental stages from 2-cell embryos (Mitalipova *et al*., 2001) up to Day 12 hatched blastocysts (Gjørret and Maddox-Hyttel, 2005). However, even though ESC-like cell lines were established and some of them could be cultured for extended periods of time, their characterization, especially with respect to functional contribution to chimeras, remained obscure. At present, it must be concluded that none of the derived cell lines have been capable of contributing to germline transmitting chimeras (Iwasaki *et al*., 2000) and, thus, can not be classified as *bona fide* ESCs. A very recent breakthrough indicates that a combination of FGF2 and an inhibitor of the canonical Wnt-signaling pathway may be the key to maintain bovine ESCs (Bogliotti *et al*., 2018).

Similar activities materialized in the pig where we and many others attempted to establish porcine ESCs (for review, see Telugu *et al*., 2010; Ezashi *et al*., 2016). However, even though a single report on a porcine ESC-derived chimera is found (Chen *et al*., 1999), none of the derived cell lines were capable of contributing to germline transmitting chimeras. Interestingly, cells from the inner cell mass from Day 6 to 7 porcine blastocysts are capable of contributing to such germline transmitting chimeras (Anderson *et al*., 1994; Onishi *et al*., 1994; Nagashima *et al*., 2004), and are, by this criterion, pluripotent and hereby a potential source of ESCs. Clearly, however, such is pluripotent cells loose this potential when cultured for even a short period of time. More recent data, where porcine ESCs again have been demonstrated to give rise to chimeric contribution, indicate that a novel medium including a combination of bFGF and LIF may represent a breakthrough although follow up with respect to germline transmission is warranted (Xue *et al*., 2016).

### ICM and epiblast differentiation in the pig

In order to explain our lack of success in deriving bovine and porcine ESC, we undertook a set of fundamental studies of the porcine ICM and epiblast which clearly demonstrated that ungulate ICM and epiblast development and pluripotency show distinct differences as compared with its murine counterpart. A dynamic change in gene expression is the driving force for the first cell differentiation, i.e. the segregation of the compacting blastomeres into the ICM and trophectoderm. In the mouse, the ICM develops a stable regulatory circuit, in which the transcription factors Nanog (Chambers *et al*., 2003; Mitsui *et al*., 2003), OCT4 (Nichols *et al*., 1998; Schöler *et al*., 1990), SOX2 (Avilion *et al*., 2003), and SAL4 (Elling *et al*., 2006; Zhang *et al*., 2006) promote pluripotency and suppress differentiation. In contrast, in the trophectoderm-destined cells, the transcription factors CDX2 and EOMES are upregulated together with ELF5 and TEAD4, which are transcription factors acting upstream of CDX2 to mediate trophectoderm differentiation (Ng *et al*., 2008; Nishioka *et al*., 2008; Yagi *et al*., 2007). On the other hand, expression of the trophectoderm-associated transcription factors, CDX2, TEAD4, and ELF5, are repressed in the ICM by the regulatory circuit of Nanog, SOX2, and OCT4 (Ralston and Rossant, 2005). In the pig, the expression of CDX2 during preimplantation development appears conserved as compared with the mouse (Kuijk *et al*., 2007). OCT4 is, on the other hand, expressed in both the ICM and trophectoderm as opposed to the mouse (Keefer *et al*., 2007; Kuijk *et al*., 2008), and Nanog expression has not been observed in the porcine ICM (Hall *et al*., 2009). Hence, there are marked species differences with respect to the molecular background for ICM and trophectoderm specification.

The embryo hatches from the zona pellucida by Days 7 to 8, and in parallel the OCT4 expression, which was earlier present in both the ICM and the trophectoderm, becomes confined exclusively to the ICM (Vejlsted *et al*., 2006), whereas expression of Nanog is still lacking (Wolf *et al*., 2011) as opposed to the mouse. At the time of hatching, the ICM separates into two distinct cell populations. Hence, the most “ventral” cell layer towards the blastocyst cavity flattens and, finally, delaminates forming the hypoblast, whereas the “dorsal” cell population establishes the epiblast. The hypoblast subsequently extends along the inside of the trophectoderm forming a complete inner epithelial lining. The polar trophectoderm covering the epiblast (Rauber’s layer) becomes very thin around Day 9 of gestation and gradually disintegrates exposing the epiblast to the uterine environment, which is very unlike the situation in the mouse, where the trophectoderm stays intact. Before the shedding of Rauber’s layer, tight junctions are formed between the epiblast cells and the adjacent trophectoderm to maintain the epithelial sealing of the embryo despite the loss of the polar trophectoderm. Apparently, the porcine epiblast forms a small cavity, which finally opens dorsally followed by an “unfolding” of the complete epiblast upon the disintegration of Rauber’s layer forming the embryonic disc (Hall *et al*., 2010). In parallel with the formation of the embryonic disc, the porcine epiblast starts to express not only OCT4, but also Nanog (Wolf *et al*., 2011b). At this stage of development, the first sign of anterior-posterior polarization develops in the embryonic disc: As mentioned earlier, the epiblast is underlaid by the hypoblast, and an area of increased cell density of closely apposed hypoblast cells develops. This area is approximately the same size as the embryonic disc, but it is dislocated about one third of its diameter anteriorly as compared with the epiblast of the embryonic disc (Hassoun *et al*., 2009; Wolf *et al*., 2011b). It is likely that this dense hypoblast region emits signals to the epiblast which suppress mesoderm-formation in the anterior epiblast regions. In this sense, the hypoblast may carry the blue-print for the specification of the epiblast.

During Days 11 to 12, the porcine embryonic disc develops into an oval shape, and a crescent-shaped accumulation of cells are found in the posterior region of the disc (Vejlsted *et al*., 2006). This crescent includes mesodermal progenitors which express the mesodermal markers, T (Brachyury) and Goosecoid (Blomberg *et al*., 2006; Wolf *et al*., 2011a), and apparently ingression of Brachyury-expressing extra-embryonic mesoderm is initiated from this crescent even before the “true” gastrulation starts with the appearance of the primitive streak (Wolf *et al*., 2011a), again, as opposed to the mouse.

With the development of the embryonic disc, a very peculiar pattern of OCT4 and Nanog expression develops in the porcine epiblast: The majority of epiblast cells express OCT4, but small groups or islands of cells are OCT4 negative (Wolf *et al*., 2011b). The latter cells, on the other hand, express Nanog resulting in a mutually exclusive expression pattern. Subsequently, Nanog expression is lost in almost the entire epiblast, except for a few cell in the most posterior region of the embryonic disc, in which OCT4 is also expressed (Wolf *et al*., 2011b). The latter cells are believed to be the primordial germ cells (PGCs).

In conclusion, the efforts on establishing bovine and porcine ESCs have been plentiful but none of them resulted in *bona fide* ESC lines that were capable of giving rise to germ line transmitting chimeric embryos. Reasons for this lack of success are probably multifactorial (Ezashi *et al*., 2016). First of all, the initial embryonic development in cattle and pig differs significantly from that in the mouse: Bovine and porcine embryos have a more protracted development of the epiblast from the inner cell mass, in contrast to the mouse, the bovine and porcine epiblast penetrates the trophectoderm (Rauber’s layer) and become exposed to the uterine environment and, finally, the bovine and porcine embryo adheres to the uterine epithelium instead of implanting through the epithelium as their murine counterpart. Second, the well-established markers of pluripotency are much less distinct and well-defined in bovine and porcine ESC-like cells than in their murine counterparts. Finally, the pluripotency states, i.e. naïve vs. primed, are not well recognized in bovine and porcine ESC-like cells. Hence, the culture conditions and needs for supplementation for maintenance of pluripotency are putative and in many studies both LIF and bFGF are used.

Importantly, in 1996 it was elegantly demonstrated that cloned sheep could be established by somatic cell nuclear transfer (SCNT) from a cultured cell line established from embryonic discs (Campbell *et al*., 1996). This breakthrough later led to the birth of Dolly (Wilmut *et al*., 1997), cloned from an adult mammary epithelial cell line, and to an alternative avenue for production of genetically modified large domestic species by SCNT utilizing genetically modified cell lines (Schnieke *et al*., 1997; McCreath *et al*., 2000). With this development, the practical importance of bovine and porcine ESCs became less evident as seen in an agricultural and biomedical perspective.

## Third medical phase: Human induced pluripotent stem cells as models for neurodegeneration

Through our struggles towards establishing bovine and porcine ESCs, we developed a skill set that allowed us to embark on human iPSCs and the use of these fascinating cells for modelling neurodegeneration. Eminent funding opportunities prompted us to move from an agricultural focus into the medical arena.

### Human iPSC reprogramming and mesenchymal-to-epithelial transition

In 2006, Takahashi and Yamanaka published their conceptual work on the establishment of murine iPSCs, where they elegantly narrowed down the need of reprogramming factors to the so-called Yamanaka factors: *Oct4, Sox2, Klf4* and *c-Myc* (OSKM), which were introduced by retroviral vectors (Takahashi and Yamanaka, 2006). Only one year later, two groups independently reported on the establishment of human iPSCs (Takahashi *et al*., 2007; Yu *et al*., 2007). Since these first publications a range of iPSC reprogramming technologies have been developed and refined with respect to both reprogramming factors (gene sequences, mRNA, miRNA, protein) and vectors (integrating and non-integrating viruses, minicircle vectors and episomal plasmids) in combination with different epigenetic modifiers (for review, see Malik and Rao, 2013). As the most novel approach, it has been demonstrated that the use of CRISPR transcriptional activators for prompting endogenous pluripotency gene expression can result in iPSC reprogramming (Weltner *et al*., 2018).

We have refined and characterized a non-integrative episomal plasmid-based human iPSC reprogramming strategy first published by Okita *et al*. (2011). Our reprogramming is based on the use of electroporation of fibroblasts with three plasmids encoding a short hairpin to TP53 (*shp53*) combined with human *OCT4, SOX2, KLF4, L-MYC* and *LIN28*. We have clearly demonstrated that this strategy, including transient p53 suppression, increases reprogramming of human fibroblasts without affecting apoptosis and DNA damage (Rasmussen *et al*., 2014). Moreover, we have performed a detailed investigation of the gene expression and ultrastructural changes associated with the mesenchymal-to-epithelial transition (MET) that is a vital component of the iPSC reprogramming process (Høffding and Hyttel, 2015). We clearly demonstrated that the sequential acquisition of an epithelial epiblast-like ultrastructure was accompanied by a reorganization of actin and beta-catenin localization from the cytoplasm to the plasma membrane region as well as appearance of plasma membrane-associated E-cadherin and Occludin and of Nanog in the nucleus. In parallel, the mesenchymal marker vimentin disappeared. At the transcriptional level, the relative expression of the epithelial markers *CDH1, OCLN* and *EPCAM* was, accordingly, dramatically increased through MET. On the other hand, transcription of the mesenchymal markers *VIM, ZEB1* and *SLUG* appeared constant or slightly downregulated. The true downregulation was probably masked by the large number of non-reprogrammed fibroblasts in the samples. These studies clearly demonstrated that a well-orchestrated MET is a major component of iPSC reprogramming.

The investigations referred to above gave us a solid platform for iPSC-based disease modelling, which was materialized in the stem cell center of excellence in neurology, BrainStem.

### Human iPSCs for modelling neurodegeneration

The iPSC technology gives access to an infinite source of pluripotent cells from an individual, offering great potentials for future disease modelling and cell therapy (Condic and Rao, 2010). *In vitro* disease modelling has become a major tool in the potential identification of novel disease phenotypes and drug targets as well as in drug screening. Worldwide, iPSCs are used for modelling a variety of disorders, but they are especially useful in research focusing on late progressive disorders such as neurodegenerative diseases, like frontotemporal dementia (FTD), amyotrophic lateral sclerosis (ALS), Alzheimer’s (AD) and Parkinson’s disease (PD), where early symptomatic brain samples are impossible to obtain (Hargus *et al*., 2014; Hedges *et al*., 2016; Lee and Huang, 2017). The iPSC technology allows for creation of “micro-brains” in a dish and studies of the specific pathology and disease progression in an easily assessable and manipulated environment.

Previously, research in the underlying mechanisms of neurodegeneration, as e.g. AD, has been based on data from transgenic AD mice models, which are unfaithful in mimicking AD, or post-mortem AD brain tissue, which exclusively represents the terminal disease pathology. These shortcomings are a major setback for the development of novel therapeutics, which need to combat early disease progression. The iPSC technology, on the other hand, offers the opportunity to investigate early disease mechanisms in the relevant targets: The human neurons, astrocytes and microglia. An example of modelling of frontotemporal dementia (FTD) is presented in the following as it encompasses all components of stem cell biology and gene editing required for dissecting early disease mechanisms.

Frontotemporal dementia linked to chromosome 3 (FTD3) is a rare heterozygous early-onset form of frontotemporal dementia, which is caused by a point mutation in the gene encoding the charged multivesicular protein 2B (CHMP2B) located to the human chromosome 3. FTD3 is characterized as a behavioral variant of frontotemporal dementia mainly associated with initial mild personality changes and social inabilities and as the disease commences potential development of apathy and aggressive behavior (Seelaar *et al*., 2011). FTD3 slowly progresses from the age of onset around the late 50’ties with a mean duration of approximately 10 years, and is thus defined as an early-onset form of dementia (Isaacs *et al*., 2011; Rossor *et al*., 2010; Tang *et al*., 2012). The Danish version of FTD3 has spread in a large family and is caused by a single nucleotide mutation translated into shortened and altered C-terminus of the CHMP2B protein (Skibinski *et al*., 2005; Urwin *et al*., 2010; Zhang *et al*., 2017). CHMP2B is an important part of the endosomal sorting complex required for transport-III (ESCRT-III) and for a proper function of the intracellular endolysosomal pathway (Krasniak and Ahmad, 2016; Urwin *et al*., 2010; van der Zee *et al*., 2008). The mutation results in truncated CHMP2B unable to mediate the endosomal-lysosomal fusion and processing. Patient-derived iPSCs have proven to be very useful in identification of cellular and molecular FTD3 phenotypes and future studies utilizing such models will likely reveal potential therapeutic targets. The stepwise process of FTD3 disease modelling is presented in the following (Zhang *et al*., 2017).

Skin fibroblasts were harvested from the patients and reprogrammed into iPSCs as by means of the non-integrative episomal plasmid approach described above (Rasmussen *et al*., 2014). Before using the iPSC lines for experimentation, they were carefully characterized with respect to their expression of pluripotency markers, ability to differentiate into all three germ layers in-vitro, normality of karyotype and absence of episomal plasmids in their genome.

For disease modelling of neurons, the iPSCs were submitted to neural induction via a dual SMAD inhibition using the small molecules SB431542 and LDN193189, which inhibits the TGFβ and the BMP pathway, thus promoting ectodermal and neuronal differentiation, respectively (Zhang *et al*., 2017). The maturation of the neuronal progenitor cells into glutamatergic forebrain cortical neurons was initiated and maintained with growth factor supplements of BDNF, GDNF and the γ-secretase inhibitor DAPT (Zhang *et al*., 2017).

Until recently, reference iPSCs were derived from healthy age- and gender matched control individuals and used as a comparison to the iPSCs derived from the patients. With the introduction of clustered regularly interspaced short palindromic repeats (CRISPR)-based gene editing, it is now possible to create isogenic controls from the patient’s own cells to use as a reference instead, eliminating obvious bias due to genomic variance (Poon *et al*., 2017). This so called CRISPR/Cas9 technology is derived from a natural adaptive immune defence mechanism in bacteria providing protection against DNA sequences invading from bacteriophages (Rath *et al*., 2015). Today, this microbial immune mechanism has been biotechnologically transformed into a versatile tool for genome editing. Hence, a single stranded guide-RNA sequence (sgRNA), designed to recognize a specific site in the genome, is combined with a Cas9 protein, capable of cleaving double stranded DNA. Once the target DNA is cut by Cas9 by a double stranded break, the cell repairs the break by either non-homologous-end-joining (NHEJ) or homology-directed repair (HDR) (Ran *et al*., 2013). NHEJ, which is by far the most common of the two, is a random default-prone mechanism where the DNA-recombinase repairs the break by adding random nucleotides until the two DNA strands once again are connected. NHEJ is likely to result in insertions or deletions (Indels), which often results in formation of a codon shift creating a premature stop codon. NHEJ can, however, be bypassed by HDR where an alternative, often single stranded, DNA template carrying a designed sequence with overhangs matching the DNA regions beside the cut is provided. This oligo will, in successful cases, function as a template for DNA-repair (Hsu *et al*., 2014; Ran *et al*., 2013; Yumlu *et al*., 2017). In the case of FTD3, isogenic controls were created in three different patients (Zhang *et al*., 2017).

Based on the use of patient iPSC-derived neurons and their isogenic controls, we have clearly demonstrated specific disease phenotypes in the FTD3 neurons, all of which can be rescued by correction of the disease-causing mutation. These include mis-regulated expression of genes related to endosomes, mitochondria and iron homeostasis, which was verified by immunocytochemistry, electron microscopy and cellular assays demonstrating large neuronal accumulations of endosomes, lack of mitochondrial axonal distribution and cristae formation, reduction in mitochondrial respiration capacity and intracellular iron accumulation (Zhang *et al*., 2017). Further studies in disease modelling of FTD3 using iPSCs will potentially reveal additional novel disease phenotypes and therapeutic strategies.

The central nervous system holds a glial/neuron ratio of 1.48 (Friede and Van Houten, 1962; Sica *et al*., 2016), which emphasize the glial importance and points towards potential pathological implications of glia in neurodegenerative disorders like FTD. Consequently, we applied an astrocyte differentiation protocol where growth factor supplements, mimicking *in vivo* embryonic astrogenesis, promoted the differentiation and maturation of the neuronal progenitor cells into astrocyte progenitors and further towards astrocytes expressing the astrocytic markers AQP4, S100β, SOX9 and GFAP (unpublished data). Our studies of FTD3-derived astrocytes and their isogenic controls clearly demonstrated that the FTD3 astrocytes displayed accumulation of autophagosomes and increased astrocyte reactivity with a subsequent toxic effect on neurons (unpublished data). Hence, not only neurons, but also the prominent glial compartment is affected by FTD3. Continued research on co-cultures between neurons and glial cells, including both astrocytes and microglia, will further aid unravelling the molecular mechanisms behind this autophagic imbalance and induced neurotoxicity.

## Fourth biomedical and veterinary phase: Porcine and canine iPSCs and canine cognitive dysfunction

The competences gained from the human iPSC-based disease modeling allowed us to return our focus to studies of porcine and canine iPSCs of more biomedical, veterinary and, potentially, agricultural relevance.

### Porcine iPSCs

Porcine iPSCs have attracted great attention due to the fact that pigs are excellent biomedical models where potentials, but also risks associated with iPSC-based therapy may be investigated. The use of this model enables long-term studies of, for example, cell or organ transplantation, and a multitude of genetically modified pigs are emerging as models for human diseases (Perleberg *et al*., 2018). In addition to being used for modeling cell-based therapy, porcine iPSCs may also facilitate the generation of genetically modified pigs for use as preclinical models and, potentially in the future, production of animals with valuable traits through the use of chimeric or nuclear transfer technologies. For these reasons we set out to derive integration-free porcine iPSCs.

As alluded to earlier, *bona fide* porcine ESCs have not been generated (Gandolfi *et al*., 2012). The derivation of iPSCs, therefore, is of great importance, and at least 25 studies have already described putative porcine iPSC production (for review, see Pessôa *et al*., 2019). The production of porcine iPSCs until now has predominantly utilized integrative viral vectors carrying human or murine *OCT4, SOX2, KLM4* and *C-MYC*, including some variations such as *NANOG* and *LIN-28*. However, persistent expression of the integrated transgenes has been widely reported, as opposed to the mouse, and failure to inactivate the exogenous factors is considered a major flaw in the generation of *bona fide* porcine iPSCs (Ezashi *et al*., 2016).

Contribution of porcine iPSCs to live chimeric offspring and germline transmission has only been achieved by one group thus far (West *et al*., 2010; West *et al*., 2011). In this study, porcine mesenchymal stem were used for the iPSC reprogramming and this approach resulted in more than 85% of the live-born piglets being chimeras. Interestingly, this approach also allowed for germline transmission where 2 out of 43 next generation piglets were of iPSC-origin. One of these piglets was, however, stillborn and the other only lived to Day 3 indicating that underlying potential epigenetic aberrancies are incurred.

As for the putative porcine ESCs, the pluripotency state, i.e. naïve vs. primed, of the porcine iPSCs has remained elusive and unclarified. Interestingly, the porcine iPSCs giving rise to germline transmission were cultured in the presence of bFGF being typical for primed murine ESCs, which are not capable of giving rise to germline transmitting chimeras (West *et al*., 2010; West *et al*., 2011). Again, this underlines the lack of clarity regarding the pluripotency states in the pig.

We have particularly focused on the derivation of integration-free porcine iPSCs according to the protocol we optimized for human iPSC reprogramming (Rasmussen *et al*., 2014). Porcine iPSCs were successfully generated by this methodology and cultured in the presence of bFGF as well as MEK/ERK (PD0325901) and GSK-3β (CHIR99021) inhibitors (Li *et al*., 2018). In order to assess the transgene status with respect to genomic integration or plasmid persistence in our iPSCs, PCR analysis on total DNA extractions, which included genomic DNA and episomal plasmid DNA, were performed. These revealed that at least two of the three episomal plasmids were still present in all lines examined at passage 10. However, at passage 20 the abundance of the two plasmids was significantly diminished in all iPSC lines with one plasmid being completely undetectable. This promoted us to select the a porcine iPSC line, which showed the weakest PCR products for the two plasmids, for single cell subcloning under the assumption that the cell line might show a certain diversity with respect to plasmid integration or retention. Indeed, 6 out of 8 subclones were completely free of episomal vector DNA. We hereby succeeded in generating porcine iPSCs free of the reprogramming constructs. One of the most striking findings during this quest was that subcloning appears to be crucial in order to obtain integration- and episomal-free porcine iPSCs using the plasmid approach.

During our efforts in implementing the plasmid-based iPSC reprogramming in the pig, we discovered a small population of stage-specific embryonic antigen 1 positive (SSEA-1+) cells in Danish Landrace and Göttingen minipig embryonic fibroblasts, which were absent in their Yucatan counterparts (Li *et al*., 2017). Interestingly, reprogramming of the SSEA-1+ cells after cell sorting led to higher reprogramming efficiency. These SSEA-1+ cells exhibited expression of several genes that are characteristic of mesenchymal stem cells.

### Canine iPSCs

Dogs are considered as very interesting models for human diseases; not only due to the over 200 hereditary canine diseases with equivalents in humans, but also due to the physiological similarities as well as equivalence in response to therapy (Starkey *et al*., 2005; Gilmore and Greer, 2015). Based on the competences gained from our human iPSC modelling of neurodegeneration, we extended our studies back to the veterinary field focusing on the dog. Recently, the neurobehavioral syndrome canine cognitive dysfunction (CCD), which shares many clinical and neuropathological similarities with human aging and early stages of AD, has been characterized in dogs, and it is increasingly evident that humans and dogs demonstrate commonalities in brain aging associated with cognitive dysfunction (Studzinski *et al*., 2005; Cotman and Head, 2008). The prevalence of CCD in dogs over 8 years of age has been estimated to 14.2-22.5% (Azkona *et al*., 2009; Salvin *et al*., 2010). Hence, we set out to further characterize the CCD condition in iPSC-derived neurons from aged demented and control dogs, which will also allow the comparison of CCD with human AD at the cellular level. Such studies have several perspectives: The dog may in the future serve as a model for spontaneous AD in humans and from a veterinary point of view, novel treatment modalities of CCD may become available.

The first information on potential canine iPSCs was reported some years after Yamanaka’s breakthrough (Takahashi and Yamanaka, 2006), and the quest for deriving fully reprogrammed and stable canine iPSCs is still ongoing (Shimada *et al*., 2010; Lee *et al*., 2011; Luo *et al*., 2011; Whitworth *et al*., 2012; Koh *et al*., 2013; Baird *et al*., 2015; Nishimura *et al*., 2017; Gonçalves *et al*., 2017; Chow *et al*., 2017; Tsukamoto *et al*., 2018). In the first studies on canine iPSCs, canine reprograming factors were utilized for reprogramming (Shimada *et al*., 2010). The presumptive iPSCs were positive for OCT4 and alkaline phosphatase and were capable of directed differentiation into representatives of all three germ layers. However, the cells were not extensively characterized. In the subsequent work, researchers used mostly human or mouse OSKM reprogramming factors, occasionally with addition of *LIN28* and *NANOG* (Whitworth *et al*., 2012), introduced using retroviral (Shimada *et al*., 2010; Koh *et al*., 2013; Baird *et al*., 2015) or lentiviral approaches (Lee *et al*., 2011; Luo *et al*., 2011; Whitworth *et al*., 2012; Nishimura *et al*., 2017; Gonçalves *et al*., 2017). Lastly, non-integrative Sendai virus have been attempted (Chow *et al*., 2017; Tsukamoto *et al*., 2018). Again, as earlier described for the pig, the silencing of the integrated transgenes in the canine iPSCs seems to represent a consistent problem and was only described in a few studies (Baird *et al*., 2015; Gonçalves *et al*., 2017). Regarding culture conditions and supplementation requirements, canine iPSCs seem to be dependent of both LIF and bFGF, with some exceptions (Whitworth *et al*., 2012; Nishimura *et al*., 2017; Gonçalves *et al*., 2017; Chow *et al*., 2017), as well as the cells are dependent on culture with feeder cells, except for a single report (Nishimura *et al*., 2017).

In general, the reports on putative canine iPSCs do not refer to the naïve vs. primed pluripotency state of the generated cells. However, based on the expression of pluripotency markers, one can speculate that most of the generated cell lines represent a primed status, characterized by expression of markers such as SSEA4, TRA-1-60 and TRA-1-80 (Lee *et al*., 2011; Luo *et al*., 2011; Whitworth *et al*., 2012; Baird *et al*., 2015; Nishimura *et al*., 2017; Chow *et al*., 2017). Nevertheless, canine iPSCs expressing naïve pluripotency markers, like SSEA1, have also been described (Koh *et al*., 2013; Tsukamoto *et al*., 2018). The expression of pluripotency markers, however, may differ between species making it difficult to draw firm conclusions on the state of pluripotency just based upon such markers. Overall, the potential canine iPSCs have been reported to show different combinations of classic pluripotency markers, such as OCT4, Nanog, SOX2, amongst others (for review, see Pessôa *et al*., in press), and some of these cells lines were also able to form teratomas (Lee *et al*., 2011; Whitworth *et al*., 2012; Koh *et al*., 2013; Gonçalves *et al*., 2017; Chow *et al*., 2017; Tsukamoto *et al*., 2018). Contribution of iPSCs to the development of chimeric embryos has, however, not been described so far.

All the previously cited reports deal with reprogramming of fibroblasts or adipose tissue cells from canine embryos, fetuses or younger adults, the oldest donors being 3-year-old beagles and a 6-year-old male standard poodle (Koh *et al*., 2013; Chow *et al*., 2017; respectively). Our studies of CCD focused on geriatric dogs, and it turned out that iPSC reprogramming of fibroblasts from such elderly dogs is a major challenge. Our efforts, however, have just started to pay off. We have attempted to reprogram adult fibroblasts to pluripotency using an excisable lentiviral vector containing human and/or murine OSKM (Sommer *et al*., 2009; Gonçalves *et al*., 2017). After a longer series of experiments, the first iPSCs colonies have now emerged around 14 days after transduction with human factors in skin fibroblasts of a 14-year and 9 month-old female west highland white terrier. So far, colonies obtained are flat, present high nuclei to cytoplasm ratio, are tightly packed, present well defined edges, and are positively stained for alkaline phosphatase and hanog The potential iPSCs are dependent on both LIF and bFGF and are in the process of expansion for further characterization. Once these cell lines are well stablished and characterized, we hope they will provide valuable information for iPSC-based disease modeling and veterinary research.

## Fifth agricultural phase: Return to bovine *in vitro* embryo production

This review began with *in vitro* production of bovine embryos. Even though great advances were made in this area in Denmark during the eighties and nineties, the practical implementation of the technologies failed due to concerns related to animal welfare and ethical considerations related to the OPU procedure and the risk of LOS. As alluded to earlier in the text, the refinement of media for bovine IVP has more or less eliminated the risk for LOS, and the OPU procedures have also become less harmful. These developments have led to a Danish reconsideration of the use of the technologies in cattle breeding and have given leverage to funding of the project EliteOva by Innovation Fund Denmark. EliteOva aims at implementing OPU and IVP combined with genomic selection of the embryos in commercial Danish Holstein dairy breeding.

It has been a great privilege to encompass a full circle of scientific progress from bovine oocytes and embryos through bovine and porcine stem cells into human stem cell-based disease modeling and back to animal stem cells and, finally, practical implementation of bovine oocyte and embryo technologies.

## Conclusions

The veterinary and animal science professions are rapidly developing and their inherent and historical focus on agriculture has been extended with a major biomedical and even medical dimension. This biomedical and medical trend in science cannot be rejected and should be contemplated as an opportunity for contemporary development of the veterinary and animal science professions. With an open scientific mind it is possible to embark on such biomedical and medical adventures and gain new competences that can feed back to novel ideas and projects in the veterinary and animal science field. Hence, seek opportunistic scientific avenues and see the possibilities in gaining novel competences that will, in turn, benefit veterinary and animal science.
